# Case Report: Anti-cavin-4-positive immune-mediated rippling muscle disease co-occurring with isolated ocular myasthenia gravis

**DOI:** 10.3389/fimmu.2026.1762980

**Published:** 2026-08-05

**Authors:** Baitong Wang, Anzhe Zheng, Mingshi Gao, Dongyue Yue, Wenhua Zhu, Zhiguo Lv, Jianying Xi

**Affiliations:** 1 Department of Encephalopathy, The Affiliated Hospital to Changchun University of Chinese Medicine, Changchun, Jilin, China; 2 Huashan Rare Disease Centre and Department of Neurology, Huashan Hospital, Shanghai Medical College, National Centre for Neurological Disorders, Fudan University, Shanghai, China; 3 Department of Pathology, Huashan Hospital, Fudan University, Shanghai, China; 4 Department of Neurology, Renji Hospital, Shanghai Jiao Tong University School of Medicine, Shanghai, China

**Keywords:** case report, comorbidity, immune-mediated rippling muscle disease, immunotherapy, myasthenia gravis

## Abstract

Immune-mediated rippling muscle disease (iRMD) is a rare autoimmune myopathy characterized by mechanically triggered, wave-like skeletal muscle contractions. Its coexistence with myasthenia gravis (MG) has been described, but detailed clinicopathological and longitudinal characterization of anti-cavin-4-positive iRMD coexisting with isolated ocular MG remains limited. Here, we report a 35-year-old man who developed painless, water-like rippling movements of the right thigh in 2023, followed by occasional palpitations and mild creatine kinase elevation in 2024. In July 2025, he developed fluctuating right ptosis without diplopia, bulbar symptoms, or limb weakness. Serology showed anti-acetylcholine receptor antibody positivity and high-titer anti-cavin-4 antibodies. Electromyography revealed mild myogenic changes with electrically silent rippling activity. Muscle biopsy showed mild myopathic changes and mosaic caveolin-3 expression. Chest CT showed no thymic hyperplasia or thymoma, and cardiac evaluation showed no structural heart disease or clinically significant arrhythmia. These findings supported anti-cavin-4-positive iRMD coexisting with isolated AChR-positive ocular MG. At follow-up, the patient reported reduced rippling activity, preserved limb strength, and persistent mild fatigue-related ptosis. Among the cases identified in our search, this was the youngest documented case with isolated ocular MG and the first anti-cavin-4-positive ocular-MG case with muscle-biopsy documentation. The case extends the documented age and mild-severity spectrum of iRMD associated with MG or AChR antibody positivity.

## 1 Introduction

Myasthenia gravis (MG) is an antibody-mediated autoimmune disorder of the neuromuscular junction, most commonly caused by acetylcholine receptor (AChR) antibodies. These antibodies impair synaptic transmission through complement activation, receptor internalization, and structural remodeling of the postsynaptic membrane. Dysregulated T-cell help, aberrant thymic selection, and B-cell hyperactivity contribute to the breakdown of self-tolerance, with thymic hyperplasia or thymoma frequently observed. Clinically, MG presents with fluctuating weakness and diurnal variation, typically improving with cholinesterase inhibitors.

In contrast, immune-mediated rippling muscle disease (iRMD) is a recently defined autoimmune myopathy characterized by muscle membrane hyperexcitability. The discovery of anti-caveolae-associated protein-4 (cavin-4) antibodies has enabled clear distinction between iRMD and hereditary rippling muscle disorders. Cavin-4, together with Caveolin-3 (Cav-3), plays a key role in stabilizing T-tubule architecture and regulating excitation-contraction coupling. Antibody-mediated disruption of this complex produces mechanically triggered rippling, wave-like contractions, and mild-to-moderate CK elevation. Since anti-cavin-4 antibodies were identified in 2022, iRMD has been increasingly recognized as part of the broader autoimmune myopathy spectrum.

Although both MG and iRMD are antibody-associated neuromuscular disorders, their coexistence remains exceptionally rare. MG-iRMD overlap may create diagnostic challenges in patients with both fatigable weakness and mechanically triggered rippling activity. Here, we describe a 35-year-old man with high-titer anti-cavin-4-positive iRMD coexisting with isolated AChR-positive ocular MG. By contextualizing this case within the existing literature, we refined the documented age and clinical-severity spectrum of iRMD-MG overlap and highlighted the practical diagnostic clues for recognizing cavin-4-related muscle autoimmunity in patients whose MG remains ocular.

## 2 Case presentation

A 35-year-old man presented to Huashan Hospital, Fudan University on 31 July 2025 with “rippling muscle activity for over two years and drooping of the right eyelid for over one month.” He had no relevant personal or family history of autoimmune, neuromuscular, malignant, toxic, or statin-related disease. The clinical timeline is summarized in **Supplementary Table 3**.

### 2.1 Early phase: rippling activity and subtle enzymatic abnormalities

In 2023, the patient noticed water-like undulating movements in his right thigh muscles after rest or during light exertion. The movements were painless and not accompanied by weakness, atrophy, or rigidity. Owing to minimal functional impact, he did not seek medical care.

In 2024, he developed occasional palpitations, though without typical cardiac symptoms such as chest tightness, chest pain, or dyspnea. Routine testing revealed mildly elevated creatine kinase (CK, 270 U/L; reference 34–174), indicating subclinical myofibre injury, but no intervention was initiated.

### 2.2 New symptoms: ocular ptosis and diagnostic reevaluation

In July 2025, he developed right eyelid ptosis showing diurnal fluctuation—mild in the morning, worsening by evening. He denied experiencing diplopia, dysphagia, dysarthria, or limb weakness. At the local hospital, pyridostigmine bromide was prescribed at 120 mg three times daily for suspected myasthenia gravis. However, neither the right ptosis nor the rippling muscle activity showed appreciable improvement.

Because of the poor response, he sought further assessment at Huashan Hospital. The long-standing rippling movements and CK elevation raised suspicion of a concurrent myogenic disorder. Comprehensive investigations were arranged.

### 2.3 Precise diagnostic workup

**Figure 1:**
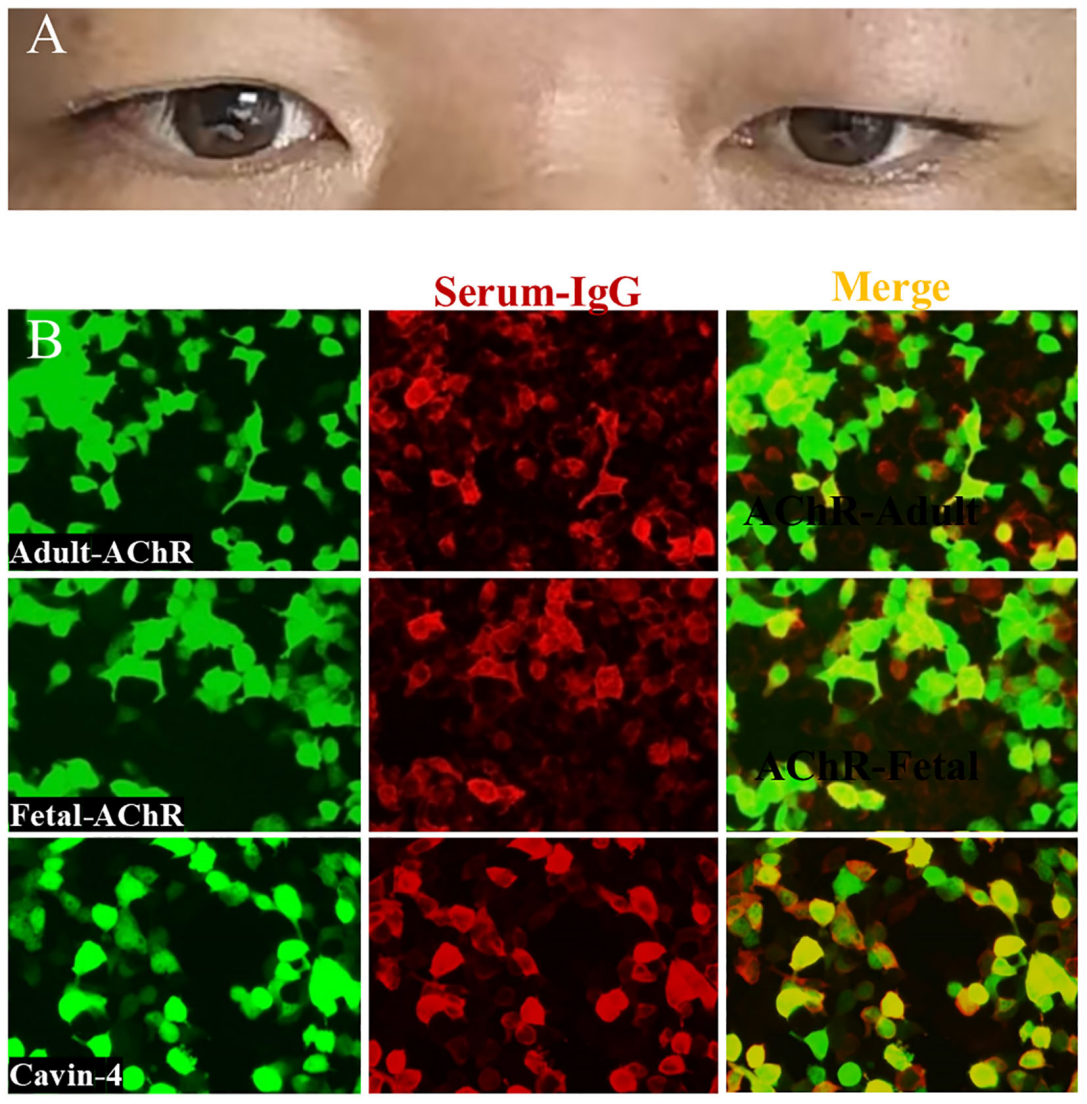
**(A)** Incomplete right ptosis in the patient. **(B)** Upper panel: Positive anti-adult acetylcholine receptor (AChR) IgG antibody, with a titer of 1:10; Middle panel: Positive anti-fetal acetylcholine receptor (AChR) IgG antibody, with a titer of 1:30; Lower panel: Positive anti-caveolae-associated protein-4 (cavin-4) IgG antibody, with a titer of 1:300.

On admission, he was alert, oriented, and hemodynamically stable. Neurological examination revealed incomplete right ptosis (palpebral fissure 6 mm on the right vs 8 mm on the left, **Figure 1**) and visible bilateral rippling movements in the thighs after percussion or stretching (**Supplementary Video**). Ocular motility and pupillary reflexes were intact. Muscle strength was Grade V throughout, and reflexes were brisk but symmetrical. Sensory and cerebellar findings were normal.

Routine biochemistry showed modest elevations in CK (319 U/L) and alanine aminotransferase (89 U/L). Other laboratory findings are detailed in **Supplementary Table 1**.

Chest CT excluded thymic hyperplasia or thymoma. Abdominal ultrasound revealed fatty liver. Cardiac evaluation by ECG and 24 hour holter in March 2025, approximately five months before the index neuromuscular admission, showed no clinically significant arrhythmia, although first-degree atrioventricular block, right-axis deviation, and incomplete right bundle branch block were documented. Echocardiography showed normal chamber dimensions and wall motion with LVEF 63% (**Supplementary Table 1**).

Motor and sensory nerve conduction studies were normal. Repetitive nerve stimulation (RNS) of facial and limb muscles showed no significant decrement or post-exercise increment in Compound Muscle Action Potential (CMAP) amplitudes. Needle electromyography (EMG) demonstrated mild myogenic involvement, characterized by occasional fibrillation and narrow, polyphasic motor unit potentials with normal recruitment. During clinically visible rippling episodes, the EMG remained electrically silent, which is a hallmark feature of rippling muscle activity reflecting mechanically induced membrane instability rather than electrical hyperexcitability. This pattern is consistent with previously reported rippling muscle syndromes.

Since the patient presented with fluctuating right-sided ptosis and a negative RNS could not completely exclude ocular myasthenia gravis, serological testing was undertaken. A cell-based assay confirmed positivity for anti–acetylcholine receptor (AChR) antibodies (1:10 for adult and 1:30 for fetal AChR, **Figure 1**), while muscle-specific tyrosine kinase (MuSK) and low-density lipoprotein receptor-related protein 4 (LRP4) antibodies were negative. Given the autoimmune nature of OMG, extended serological screening for iRMD was performed and a high-titer anti-cavin-4 antibody (1:300) was revealed, establishing the diagnosis of immune-mediated rippling muscle disease (iRMD).

**Figure 2:**
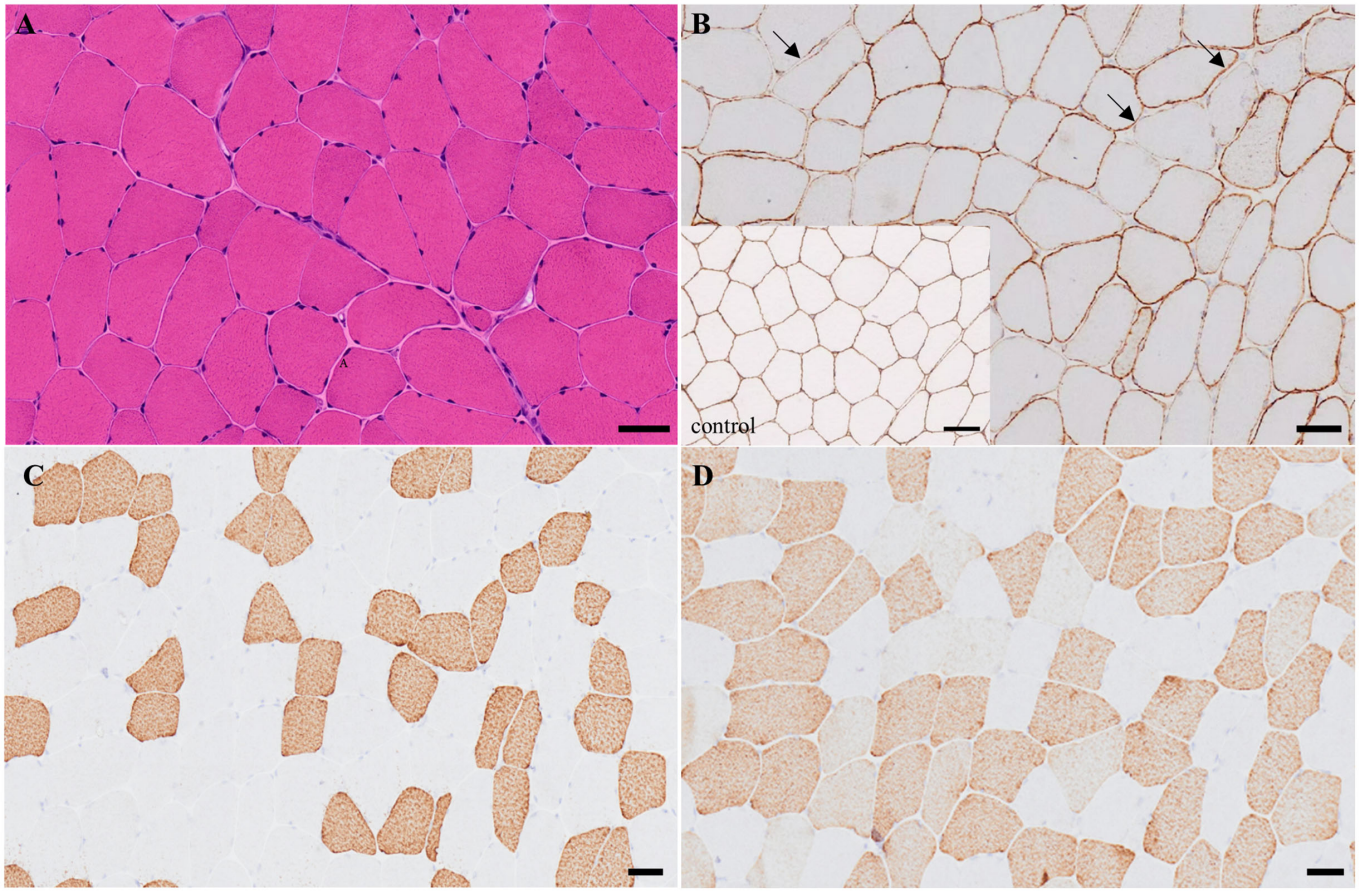
Muscle biopsy of left biceps. **(A)** Hematoxylin–eosin staining from the left biceps shows mild fiber size variability without necrosis, regeneration, or inflammatory infiltrates. **(B)** Immunohistochemistry demonstrates uneven Cav-3 expression with some fibers exhibiting weaker staining compared to adjacent fibers, resulting in a mosaic-like pattern in the patient, whereas the normal control muscle exhibits uniform and strong Cav-3 staining. **(C, D)** Myosin heavy chain type I **(C)** and myosin heavy chain IIa **(D)** staining show preserved fiber-type distribution with normal mosaic patterns and no evidence of type-specific hypertrophy or fiber-type grouping.

A biopsy of the left biceps was also performed. Hematoxylin–eosin staining showed mild atrophy and mild fiber size variability without necrosis or inflammatory infiltrates, and no type 2 fiber hypertrophy was observed. Immunohistochemistry demonstrated uneven Cav-3 expression, forming a mosaic-like pattern (**Figure 2**). In hereditary rippling muscle disease (hRMD), Cav-3 is typically absent or significantly reduced, while iRMD shows a mosaic-like pattern with uneven Cav-3 expression across muscle fibers. These immunohistochemical findings were consistent with iRMD.

### 2.4 Final diagnosis and management

The integrated clinical, serological, and pathological findings confirmed the diagnosis of ocular myasthenia gravis coexisting with immune-mediated rippling muscle disease (iRMD) for the patient. Given the shared autoimmune pathogenesis and limited efficacy of cholinesterase inhibitors in iRMD, systemic immunosuppression was chosen as the main therapeutic approach.

Taking into account the patient’s fatty liver condition and his decision to decline corticosteroid therapy, mycophenolate mofetil (0.75 g twice daily) was initiated from 11 August 2025. This conservative lower-end dose was selected because the patient had isolated ocular MG without generalized weakness and only mild CK elevation. The dose was planned to be adjusted according to clinical response and safety monitoring. Because of mildly elevated transaminases and ultrasound-confirmed fatty liver, polyene phosphatidylcholine was added for hepatic protection. He was also advised on weight control and a low-fat diet.

At an early follow-up in December 2025, the patient reported a reduction in the frequency and prominence of rippling muscle activity, with no progression of ptosis, respiratory symptoms, or generalized weakness. At updated follow-up in July 2026, he continued mycophenolate mofetil and reported preserved limb strength without generalized weakness, although very mild fatigue-related ptosis persisted. Available biochemical monitoring showed mild transaminase elevation (ALT 81.2 U/L, AST 41.6 U/L), while renal function remained within the reference range.

## 3 Literature search and case identification

To contextualize the present case, we performed a narrative literature search for reported cases of acquired or immune-mediated rippling muscle disease associated with either clinically documented MG or isolated AChR antibody positivity. PubMed was searched from database inception to 11 July 2026 using the following search strategy: (“rippling muscle disease” OR “acquired rippling muscle disease” OR “immune-mediated rippling muscle disease” OR “cavin-4” OR “anti-cavin-4” OR “caveolae-associated protein 4” OR “caveolin-3”) AND (“myasthenia gravis” OR “ocular myasthenia gravis” OR “generalized myasthenia gravis” OR “acetylcholine receptor antibody” OR “AChR antibody”). Reference lists of relevant articles and case series were also screened manually.

Cases were eligible if they provided sufficient individual-level clinical information on acquired or immune-mediated rippling muscle disease associated with either clinically documented MG or isolated AChR antibody positivity. We excluded hereditary rippling muscle disease without autoimmune overlap, non-human studies, reviews without original cases, duplicate publications, and reports lacking adequate patient-level data. Abstract-only reports were included only when sufficient individual-level information was available for extraction and were interpreted cautiously because of limited clinical, methodological, and follow-up details. Aggregate case series or cohort reports without patient-level mapping were considered qualitatively but excluded from the case-level synthesis when reliable extraction or deduplication was not possible. For descriptive analyses, eligible patients were classified into two clinically distinct groups: those with clinically documented MG and those with isolated AChR seropositivity without overt MG.

For each eligible case, we extracted demographic features, MG phenotype and MGFA classification, rippling muscle disease manifestations, comorbid thymic or malignant disease, antibody profile, biopsy findings, treatment, and clinical outcomes. This was a narrative synthesis for clinical context rather than a systematic review; therefore, no formal risk-of-bias assessment or meta-analysis was performed (**Supplementary Table 2**).

## 4 Results of literature synthesis

The narrative literature search identified 26 unique patients with sufficient individual-level information for case-level extraction and reliable deduplication. (1–15) These patients had acquired or immune-mediated rippling muscle disease associated with either clinically documented MG or isolated AChR antibody positivity. One patient was reported in two publications and was counted only once in the synthesis (1, 2). Sex was reported in 23 cases, including 19 male and 4 female patients, while sex was unavailable in 3 cases. The mean reported age was 58.2 ± 13.9 years. Clinical MG was documented in 23/26 cases, including generalized MG in 17/26 cases, ocular MG in 4/26 cases, and clinical MG with an unspecified ocular versus generalized phenotype in 2/26 cases. The remaining 3/26 patients had isolated AChR antibody positivity without overt clinical MG and were analyzed separately from the clinical MG cases. Among the 17 patients with generalized MG, 12 were classified as MGFA IIa, 2 as IIb, and 2 as IIIb, while MGFA subclassification was unavailable in 1 case.

All 26 reported cases were AChR antibody-positive. Anti-cavin-4 testing was available in 6/26 unique patients, all of whom were positive. For the AChR-seropositive iRMD patient originally reported by Liewluck et al. in 2012, cavin-4 seropositivity was retrospectively established when Dubey et al. explicitly identified the same individual as patient 6 in their 2022 cohort (1, 2). However, testing was selectively performed in clinically suspected iRMD cases. All six selectively tested patients were positive for anti-cavin-4 antibodies; however, this observation is subject to substantial ascertainment bias and should not be interpreted as an estimate of prevalence or the true positivity rate in iRMD. Among these 6 patients, 1 had ocular MG, 1 had generalized MG, 2 had clinical MG of unspecified phenotype, and 2 had isolated AChR seropositivity without overt MG.

Confirmed thymoma was reported in 3/26 cases, and 1 additional patient had a mediastinal mass of unknown pathology.

## 5 Discussion

Here we detailed described the characterization of the overlap phenotype of iRMD and MG in a relatively young adult. In this patient, the concordance of high-titer anti-cavin-4 antibodies, mechanically induced electrically silent rippling, and mosaic Cav-3 expression provided multimodal support for the diagnosis of iRMD, despite preserved limb strength and only mildly elevated CK levels. Although repetitive nerve stimulation was negative, the diagnosis of ocular MG was supported by fluctuating ptosis and AChR antibody positivity on a cell-based assay. This case therefore expands the age and clinical-severity spectrum of anti-cavin-4-positive iRMD-MG overlap and provides a practical diagnostic clue for recognizing cavin-4-related muscle autoimmunity in patients whose MG remains ocular. Among previously reported ocular MG cases with documented cavin-4 testing, this is the only one accompanied by muscle biopsy evidence.

MG and iRMD are both antibody-associated neuromuscular disorders, yet their pathological substrates differ markedly. MG is primarily a disorder of impaired neuromuscular transmission, most commonly mediated by antibodies against AChR. This leads to complement activation, postsynaptic membrane simplification, and reduced safety margin of transmission, resulting in fatigable weakness and fluctuating symptoms (16, 17). In this patient, the characteristic diurnal fluctuation of ptosis and the presence of adult and fetal AChR antibodies supported the diagnosis of ocular MG.

By contrast, iRMD involves muscle fiber hyperexcitability caused by immune-mediated disruption of structural proteins within the T-tubule system. Cavin-4, together with caveolin-3, stabilizes the T-tubule membrane during excitation–contraction coupling, and antibody binding may impair mechanosensitive channel function and intracellular calcium homeostasis (18, 19). The identification of anti–cavin-4 antibodies has provided a crucial advance in the diagnosis of iRMD in this patient, with cavin-4 now recognized as a highly specific biomarker distinguishing immune-mediated from hereditary rippling muscle disorders (1, 20).

The mechanisms underlying the coexistence of MG and iRMD remain incompletely understood. One possible explanation is epitope spreading, whereby anti-AChR-mediated junctional injury may expose additional muscle antigens and promote the development of new autoantibody specificities, including anti-cavin-4 (21). Evidence from other autoimmune myopathies supports this paradigm, where chronic muscle injury promotes diversification of autoantibody profiles. Shared immunogenetic susceptibility may also contribute to MG-iRMD overlap; however, direct genetic or HLA evidence for anti-cavin-4-positive iRMD or MG-iRMD overlap is currently lacking. Therefore, this mechanism remains speculative. A third possibility is functional interaction between cavin-4-related pathways and neuromuscular transmission. While direct structural homology between AChR and cavin-4 has not been demonstrated, cavin-4 plays a key role in stabilizing T-tubule architecture and interacts closely with caveolin-3, which is enriched at the neuromuscular junction (22). Disruption of these microdomains could theoretically alter excitation–contraction coupling, local ion-channel function, or intracellular calcium homeostasis. However, these mechanisms remain speculative, and coincidental coexistence cannot be excluded.

Treatment of coexisting MG and iRMD presents unique challenges because medications beneficial for MG may worsen iRMD symptoms. Cholinesterase inhibitors, although first-line therapy for MG, can increase synaptic acetylcholine and may exacerbate membrane hyperexcitability in iRMD, leading to worsening rippling or fasciculations. This paradoxical effect has been documented in several reports (3, 4) and was consistent with the lack of clinical improvement in our patient despite adequate pyridostigmine therapy.

Most patients with iRMD and MG respond well to immunosuppressive therapies, particularly corticosteroids, azathioprine, or mycophenolate mofetil. Selected cases may benefit from intravenous immunoglobulin (IVIG), plasmapheresis, or thymectomy. The Myasthenia Gravis Foundation of America Post Intervention Status (MGFA-PIS) outcomes often fall within minimal-manifestations or pharmacological remission, iRMD symptoms usually improve or stabilize under immunomodulatory therapy. In the present case, mycophenolate mofetil was associated with partial reduction in rippling activity and absence of progression to generalized weakness during follow-up, which contributes additional insight into therapeutic options for patients with comorbid hepatic dysfunction or steroid-related contraindications. However, very mild fatigue-related ptosis persisted. Therefore, this observation should be interpreted as partial clinical stabilization rather than evidence of complete remission or long-term therapeutic efficacy.

Given these limitations, immunosuppression forms the cornerstone of treatment for the overlap syndrome. Mycophenolate mofetil was selected for this patient due to its favorable safety profile and its ability to inhibit T- and B-cell proliferation, thereby reducing autoantibody production across multiple pathways (23). Published overlap cases were most commonly treated with glucocorticoids and/or azathioprine, while IVIG, plasma exchange, and thymectomy were used selectively (3, 4). Evidence regarding other immunosuppressive agents, including mycophenolate mofetil, remains limited in iRMD-MG overlap. For acute exacerbations of either condition, intravenous immunoglobulin and plasma exchange remain appropriate short-term interventions.

A strength of this report is the multimodal characterization integrating clinical examination, serological testing, electrophysiology, muscle pathology, and longitudinal follow-up. Nevertheless, there are several limitations for this case report. First, only Cav-3 staining was performed, and Cavin-4 staining was not included due to the unavailability of a primary antibody in our laboratory. Although high-titer serum anti-cavin-4 antibodies strongly supported the diagnosis of iRMD, lack of direct cavin-4 protein staining (primary cavin-4 antibody not available) reduced the histopathological completeness of the case. Second, single-fiber electromyography (SFEMG) was not conducted, which could have provided more detailed electrophysiological insights into the neuromuscular junction transmission. Third, the follow-up period remains relatively short. Longer longitudinal follow-up with serial clinical assessment, safety monitoring, CK measurements, and AChR and cavin-4 antibody titers is required to evaluate clinical stability, treatment safety, antibody dynamics, and the possibility of delayed thymic pathology. Finally, our literature review was narrative rather than systematic, and several reports contained incomplete information or were available only in abstract form, introducing potential reporting heterogeneity.

## 6 Patient perspective

The patient reported that the rippling movements were initially unusual but caused little functional impairment, which partly explained the delay in seeking medical attention. The subsequent development of fluctuating ptosis and the limited response to pyridostigmine prompted him to pursue further evaluation. During follow-up after initiation of mycophenolate mofetil, he perceived a reduction in the frequency and prominence of rippling muscle activity and maintained preserved limb strength, although mild fatigue-related ptosis persisted. The patient understood the need for continued follow-up, including clinical assessment, safety monitoring, CK levels, and serial AChR and cavin-4 antibody testing when available.

## 7 Conclusion

This case refines the documented age and clinical-severity spectrum of anti-cavin-4-positive iRMD–MG overlap by describing a young adult with isolated ocular MG, preserved limb strength, and only mild CK elevation, supported by concordant serological, electrophysiological, and pathological findings. Cavin-4-related iRMD should be considered when mechanically induced muscle rippling or otherwise unexplained CK elevation occurs in patients with MG.

## Data Availability

The original contributions presented in the study are included in the article/**Supplementary Material**. Further inquiries can be directed to the corresponding author.
