# Supplementary material for: Case Report: Anti-cavin-4-positive immune-mediated rippling muscle disease co-occurring with isolated ocular myasthenia gravis
Source: Front Immunol. 2026 Aug 5;17:1762980. doi: 10.3389/fimmu.2026.1762980 (PMC13485776; doi:10.3389/fimmu.2026.1762980)
Supplement: Supplementary file 1 [file Table1.docx]

Supplementary Table 1. Summary of Key Ancillary Findings

| Category | Key findings | Interpretation |
| --- | --- | --- |
| Routine biochemistry | CK 270-319 U/L ↑, ALT 89 U/L ↑, UA 0.47 mmol/L ↑ | Mild muscle injury and metabolic change |
| Inflammation markers | CRP < 0.5 mg/L, SAA < 5 mg/L | No systemic inflammation |
| Hematology | RBC 5.9 × 10¹²/L↑, Hb 170 g/L, Hct 51.6% ↑ | Mild erythrocytosis |
| Imaging | Thymus CT: no thymic hyperplasia or thymoma. | No thymic lesion |
| 12-lead ECG | Sinus rhythm, 70 beats/min; first-degree AV block, PR interval 210 ms; right-axis deviation; QRS duration 96 ms; QTc 421 ms | Mild conduction abnormality without acute ECG changes |
| Transthoracic echocardiography | Normal chamber dimensions and wall motion; LVEF 63%; TAPSE 20 mm; no significant valvular regurgitation | No structural heart disease or impaired ventricular systolic function |
| 24-hour Holter | Predominant sinus rhythm; mean heart rate 83 beats/min, range 57-120 beats/min; 2 isolated PACs and 1 isolated PVC, each <1%; no couplets or runs; longest R–R interval 1.1 s; first-degree AV block and incomplete RBBB noted | Minimal ectopic burden and no clinically significant arrhythmia |

Cardiac evaluation details: These cardiac investigations were performed before the index neuromuscular admission and were retrieved retrospectively from the patient’s prior medical records during manuscript revision.

Abbreviations: CK, creatine kinase; ALT, alanine aminotransferase; UA, uric acid; CRP, C-reactive protein; SAA, serum amyloid A; RBC, red blood cell; Hb, hemoglobin; Hct, hematocrit; CT, computed tomography; PAC, premature atrial contraction; PVC, premature ventricular contraction; QTc, corrected QT interval; TAPSE, tricuspid annular plane systolic excursion; TTE, transthoracic echocardiography; LVEF, left ventricular ejection fraction; ECG, electrocardiography; AV, atrioventricular; RBBB, right bundle branch block.

Supplementary Table 2. Summary of Reported iRMD Cases Associated With Clinical MG or Isolated AChR Antibody Positivity

|  | Publication year | Age | Sex | Country | Main symptoms  (MGFA classification) | Other manifestations | Comorbid disease(s) | Antibody type | Muscle biopsy | Treatment | iRMD Outcome | MG Outcome  (MGFA-PIS) | Reference |
| --- | --- | --- | --- | --- | --- | --- | --- | --- | --- | --- | --- | --- | --- |
| 1 | 1999 | 30 | M | Germany | Muscle rippling;  GMG (IIa) | NA | No thymoma | AChR | Nonspecific | AZA | Improved | MM-1 | [12] |
| 2 | 1999 | 55 | M | Germany | Muscle rippling;  GMG (IIb) | Myalgia,  cramps | No thymoma | AChR | NA | AZA, AChEI | Remission | Improved | [12] |
| 3 | 2004 | 38 | M | USA | Muscle rippling ;  GMG (IIa) | Hypertrophy,  myalgia | Thymoma | AChR | Nonspecific | Prednisone, plasmapheresis,  Tx, chemotherapy | Improved | Improved | [13] |
| 4 | 2006 | 54 | M | Canada | Muscle rippling ;  GMG (IIa) | Myalgia | No thymoma | AChR | Nonspecific | NA | Improved | Improved | [14] |
| 5 | 2009 | 32 | F | Germany | Muscle rippling;  GMG (IIa) | Myalgia | No thymoma | AChR | Myopathy,  caveolin-3 in mosaic pattern | Steroids, AZA | Remission | PR | [8] |
| 6 | 2009 | 57 | M | UK | Muscle rippling;  GMG (IIa) | Myalgia | No thymoma | AChR | NA | Steroids, AZA, plasmapheresis | Remission | PR | [8] |
| 7 | 2009 | 64 | M | Italy | Muscle rippling;  GMG (IIa) | Myalgia | No thymoma | AChR | Myopathy,  caveolin-3 in mosaic pattern | Steroids, AZA, IVIG | Remission | PR | [8] |
| 8 | 2009 | 67 | M | Belgium | Muscle rippling;  GMG (IIa) | Myalgia,  mounding | No thymoma | AChR | Myopathy,  caveolin-3 in mosaic pattern | Steroids, AZA | Unchanged | PR | [8] |
| 9 | 2009 | 68 | M | Germany | Muscle rippling;  GMG (IIa) | Myalgia,  mounding | No thymoma | AChR | Myopathy,  caveolin-3 in mosaic pattern | Steroids, AZA | Remission | PR | [8] |
| 10 | 2009 | 30 | M | Germany | Muscle rippling;  GMG (IIa) | Myalgia | Thymoma | AChR | Slight myopathy | Steroids, AZA | Remission | PR | [8] |
| 11 | 2009 | 53 | M | Germany | Muscle rippling;  GMG (IIa) | Myalgia | No thymoma | AChR | NA | Steroids, AZA | Remission | PR | [8] |
| 12 | 2009 | 64 | M | Netherlands | Muscle rippling;  slight OMG | Muscle stiffness | No thymoma | AChR | NA | No specific treatment | NA | NA | [15] |
| 13 | 2010 | 64 | M | UK | Muscle rippling,  GMG (IIa) | Myalgia | No thymoma | AChR | Myopathy,  caveolin-3 in mosaic pattern | No specific treatment | NA | NA | [16] |
| 14 | 2012 | 76 | F | USA | Muscle rippling,  No clinical MG; isolated AChR seropositivity | Myalgia, muscle  twitching, tightness | No thymoma | AChR, Cavin-4† | Myopathy, mosaic caveolin-3 reduction | Prednisone,  IVIG | Remission | NA | [2,17] |
| 15 | 2013 | 66 | M | Poland | Muscle rippling,  OMG | Myalgia,  stiffness | No thymoma | AChR | NA | Methylprednisolone | Improved | Improved | [18]^§^ |
| 16 | 2016 | 62 | F | Argentina | Muscle rippling,  GMG (IIb) | Myalgia | No thymoma | AChR | Myopathy,  caveolin-3 in mosaic pattern | AChEI, steroids | Improved | MM-2 | [7] |
| 17 | 2016 | 55 | M | Argentina | Muscle rippling,  GMG (IIIb) | Myalgia | No thymoma | AChR | NA | Pyridostigmine, prednisone | Improved | MM-2 | [7] |
| 18 | 2016 | 58 | M | Argentina | Muscle rippling,  GMG (IIIb) | Myalgia | No thymoma | AChR | NA | AChEI, steroids, AZA | Remission | MM-3 | [7] |
| 19 | 2022 | 63 | M | Japan | Muscle rippling,  No clinical MG; isolated AChR seropositivity | Muscle stiffness, hypertrophy, toe jerks | Mediastinal mass | AChR | Myopathy, caveolin-3 in mosaic pattern | Extended Tx | Remission | NA | [19] |
| 20 | 2022 | 51 | M | Japan | Muscle rippling,  GMG (IIa) | Myalgia | Thymoma (B2/B3) | AChR | NA | Prednisolone, pyridostigmine, Tx | Improved | Improved | [20] |
| 21 | 2022 | Mid-70s^*^ | NA^#^ | USA | Muscle rippling; MG,(MGFA NA) | Myalgia, proximal weakness, fatigue | No thymoma | AChR, Cavin-4 | Myopathy,  caveolin-3 in mosaic pattern | Prednisone, IVIG, azathioprine | Remission | NA | [2] |
| 22 | 2022 | Mid-60s^*^ | NA^#^ | USA | Lower-limb muscle rippling; OMG | Fatigue | No thymoma | AChR, Cavin-4 | NA | Prednisone | Rippling stabilized | Improved | [2] |
| 23 | 2022 | Mid-40s^*^ | NA^#^ | USA | Calf muscle rippling; MG, (MGFA NA) | Proximal weakness,  fatigue | No thymoma | AChR, Cavin-4 | Myopathy, detailed IHC findings unavailable | Prednisone, azathioprine | Remission | NA | [2] |
| 24 | 2023 | 67 | F | USA | Muscle rippling,  No clinical MG; isolated AChR seropositivity | Myalgia | No thymoma | AChR, Cavin-4 | Myopathy, reduction of caveolin-3 in mosaic pattern | Prednisone, IVIG | NA | NA | [21] |
| 25 | 2024 | 81 | M | UK | Muscle rippling,  GMG (MGFA NA) | NA | No thymoma | AChR, Cavin-4 | NA | Prednisolone, Tx | Remission | MM-1 | [22]^§^ |
| 26 | 2025 | 73 | M | USA | Muscle rippling,  OMG | NA | No thymoma | AChR | NA | No specific treatment | NA | NA | [23]^§^ |

*For age ranges reported as “mid-70s,” “mid-60s,” and “mid-40s,” ages of 75, 65, and 45 years, respectively, were used for descriptive calculations.

†Dubey et al. explicitly identified patient 6 as the previously reported 2012 case described by Liewluck et al.; cavin-4 IgG seropositivity was retrospectively established in the 2022 cohort.

#Sex was not reported at the individual-patient level for these three cases; the overall cohort comprised 7 men and 3 women.

§References [18], [22], and [23] were available only as conference abstracts and should be interpreted cautiously because of limited clinical, methodological, and follow-up details.

Abbreviations: iRMD, immune-mediated rippling muscle disease; MG, myasthenia gravis; GMG, generalized myasthenia gravis; OMG, ocular myasthenia gravis; MGFA, Myasthenia Gravis Foundation of America; NA, not available; AChR, acetylcholine receptor; AZA, azathioprine; MM, minimal manifestations; AChEI, acetylcholinesterase inhibitor; Tx, thymectomy; PR, pharmacologic remission; IVIG, intravenous immunoglobulin; Cavin-4, caveolae-associated protein 4.

Supplementary Table 3. Timeline of clinical course, diagnostic evaluation, treatment, and follow-up.

| Time | Clinical events | Investigations | Treatment / outcome |
| --- | --- | --- | --- |
| 2023 | Right thigh water-like rippling movements | Not assessed | No treatment |
| 2024 | Occasional palpitations, mild CK elevation | CK 270 U/L | No treatment |
| 12-18 March 2025 | Prior cardiac evaluation following the history of intermittent palpitations | ECG: sinus rhythm, first-degree AV block, and right-axis deviation; TTE: normal cardiac structure and LVEF 63%; Holter: sinus rhythm, heart rate 57–120 beats/min, 2 PACs and 1 PVC, without sustained arrhythmia | No specific cardiac intervention documented |
| July 2025 | Right ptosis with diurnal fluctuation | Local diagnosis of suspected ocular MG | Pyridostigmine, with poor response |
| 31 July 2025 | Initial evaluation at Huashan Hospital | Further neuromuscular investigations arranged | — |
| 11-14 August 2025 | Hospital admission and diagnostic confirmation | AChR Ab+, high-titer anti-cavin-4 Ab+, EMG silent rippling, Cav-3 mosaic pattern, and no thymic lesion | MMF 0.75 g twice daily initiated; at discharge, ptosis had partially improved but remained fluctuating, while rippling was unchanged |
| December 2025 | Early follow-up | Clinical assessment | Reduced rippling activity and stable ocular symptoms |
| July 2026 | Updated follow-up | Biochemical safety monitoring: mild transaminase elevation (ALT 81.2 U/L, AST 41.6 U/L); renal function within the reference range | Continued MMF; preserved limb strength without generalized weakness; very mild fatigue-related ptosis persisted |

Abbreviations: AChR, acetylcholine receptor; Ab, antibody; Cav-3, caveolin-3; CK, creatine kinase; EMG, electromyography; MG, myasthenia gravis; MMF, mycophenolate mofetil.
